# Editorial: Systems immunology to advance vaccine development

**DOI:** 10.3389/fimmu.2024.1527238

**Published:** 2024-12-09

**Authors:** Jue Hou, Helder I. Nakaya

**Affiliations:** ^1^ Vaccine and Infectious Disease Division, Fred Hutchinson Cancer Research Center, Seattle, WA, United States; ^2^ Department of Clinical and Toxicological Analyses, School of Pharmaceutical Sciences, University of São Paulo, São Paulo, Brazil; ^3^ Hospital Israelita Albert Einstein, São Paulo, Brazil

**Keywords:** vaccine, systems immunology and AI, vaccine development, computational biology, bioinformatics, modeling

The field of systems immunology has emerged as an essential interdisciplinary approach for understanding immune responses on a comprehensive, system-wide level. By integrating high-throughput technologies such as transcriptomics, proteomics, and computational modeling, systems immunology extends beyond traditional research methods. Systems immunology enables researchers to explore the intricate interactions within the immune components and make prediction about vaccine outcomes, accelerating the development of immunizations against pathogens, including rapidly evolving viruses like SARS-CoV-2. The studies in this Research Topic reflects these advancements, offering fresh insights into the molecular and computational aspects of immune system dynamics, which are crucial for improving vaccine efficacy.

Our Research Topic has brought together 133 authors worldwide, culminating in 16 articles showcasing cutting-edge research. These contributions cover a range of themes, from immune receptor dynamics and biomarker discovery to computational modeling and predictive analytics in vaccine responses.

## Immune receptor dynamics and antigen-specific responses

Richardson et al. explored the B cell receptor (BCR) repertoires of 40 participants from the EBL2001 clinical trial, focusing on responses to the Ad26.ZEBOV/MVA-BN-Filo Ebola vaccine. Through bulk sequencing and bioinformatic mining, the authors mapped BCR clonotypes and identified antigen-specific responses, including IGHV3-15 antibodies targeting Ebola glycoprotein, — underscoring the role of systems immunology in decoding antibody-mediated immunity. Similarly, Akhmatova et al. investigated a synthetic disaccharide conjugated to BSA (bovine serum albumin), designed to mimic Streptococcus pneumoniae serotype 3 polysaccharides. Their study demonstrated enhanced IL-17A production and γδ T cell expansion in mice, highlighting how synthetic carbohydrate-based vaccines stimulate both innate and adaptive immunity.

Haralambieva et al. explored the transcriptional profiles of B cells after a third MMR (Measles, Mumps, and Rubella) vaccine dose, identifying genes like IL20RB and BEX2 as correlates with measles-specific neutralizing antibody responses. These findings point to early biomarkers that could predict vaccine efficacy, advancing personalized vaccinology by supporting tailored vaccine schedules.

In another study, Costa-Gouvea et al. compared immune responses elicited by a *Plasmodium vivax* circumsporozoite protein malaria vaccine formulated with two different adjuvants: Poly I:C and Alhydrogel. They demonstrated that Poly I:C induced broader and stronger humoral and cellular responses, including higher levels of IgG antibodies and a more diverse IgG isotype profile, compared to Alhydrogel. The study also revealed enhanced memory B cell formation, highlighting Poly I:C’s potential to improve vaccine efficacy for malaria.

## Computational models and machine learning approaches

Several papers in this Research Topic explore computational models for designing and predicting vaccine efficacy. Khan et al. applied molecular simulations and structure-guided engineering to enhance the binding affinity of a monoclonal antibody targeting the aP2 antigen, which is linked to type 2 diabetes. Their engineered T94M mutant demonstrated superior binding strength, illustrating how computational models can advance therapeutic antibody design. Høie et al. developed DiscoTope-3.0, a computational tool that uses inverse folding latent representations to predict B cell epitopes. Benchmarked against multiple datasets, DiscoTope-3.0 excelled, particularly in predicting conformational epitopes critical for vaccine design.

In another study, Parizi et al. introduced PANDORA v2.0, a software designed to model peptide-MHC class II complexes. Their study demonstrated that PANDORA’s computational efficiency and accuracy make it a valuable tool for vaccine design and immunotherapy, especially for predicting antigenic peptides that drive immune responses. Together, these studies illustrate the transformative role of computational models in refining vaccine candidates, leveraging systems biology to predict immune outcomes and optimize antigen design.

## Biomarker discovery and vaccine reactogenicity

Carvalho et al. applied machine learning algorithms to identify baseline gene signatures associated with reactogenicity to the rVSVDG-ZEBOV-GP Ebola vaccine. By analyzing gene expression data from cohorts across four countries, the authors identified 22 critical genes associated to adverse events, offering valuable insights into how molecular profiles might predict vaccine side effects. Building on this, Martinez-Murillo et al. refined an innate plasma signature associated with the same Ebola vaccine. They identified 11 additional biomarkers, including CXCL10 and IL-15, that correlated with reactogenicity and long-term immune responses, enhancing adverse event prediction across diverse populations.

Naidu and Lulu S. investigated the immune responses to enteric infections in endemic versus non-endemic settings, finding that GRB2, a key adaptor molecule in T cell receptor (TCR) signaling, as a major immunomodulatory response in endemic regions, highlighting the importance of regional immune variations in vaccine design. This study demonstrates how systems immunology can inform the development of region-specific vaccines by identifying immune modulation mechanisms.

## T cell dynamics and antigen-specific responses

Mark et al. investigated the phenomenon of “hidden public” TCRs, which emerge following acute viral infections like lymphocytic choriomeningitis virus (LCMV) and SARS-CoV-2. Their analysis revealed that viral infections drive the expansion of shared TCRs, particularly in effector T cells, adding a new layer of understanding to how TCR repertoires function during infections. Mosmann et al. applied the SWIFT (Scalable Weighted Iterative Flow-clustering Technique) clustering algorithm to analyze intracellular cytokine staining data from the HVTN 105 HIV vaccine trial, identifying novel antigen-specific T cell populations and correlating them with antibody responses. This work provides a deeper understanding of the T cell dynamics driving vaccine-induced protection.

## Vaccine design and epitope prediction

Ali et al. used reverse vaccinology to design a multi-epitope vaccine targeting the newly identified Songling virus. By screening the viral proteome and validating epitopes through molecular docking and dynamics simulations, they identified a promising vaccine candidate with broad coverage potential. Farriol-Duran et al. introduced Brewpitopes, a bioinformatics pipeline that refines B cell epitope predictions in public health emergencies. Validated with the SARS-CoV-2 proteome, Brewpitopes achieved a fivefold enrichment in predicted neutralizing epitopes, demonstrating its potential for real-time vaccine development.

Díaz-Dinamarca et al. investigated two protein-based adjuvants, rSIP from Streptococcus agalactiae and FLH from Fissurella latimarginata, as Toll-like receptor 4 (TLR4) ligands. Their study showed that these adjuvants activate both MyD88- and TRIF-dependent signaling pathways, enhancing antigen cross-presentation and suggesting their potential as vaccine adjuvants. In another vaccine design study, Imon et al. used immunoinformatic tools to create a multi-epitope vaccine against Merkel cell polyomavirus, the causative agent of Merkel cell carcinoma. Computational simulations demonstrated strong interactions with TLR4, indicating a robust immune response, though the vaccine requires further experimental validation.

## Conclusion

The collective research presented in this Research Topic highlights the transformative potential of systems immunology in advancing vaccine development. By integrating cutting-edge techniques such as immune receptor profiling, biomarker discovery, computational modeling, and machine learning, these studies illustrate how systems immunology can unravel the complexities of immune responses. The insights gained are paving the way for more effective, personalized vaccines, improved strategies for predicting and managing adverse reactions, and the identification of novel antigenic targets. As systems immunology continues to evolve, it will remain a cornerstone in addressing global health challenges, enabling the development of next-generation vaccines that are more precise, adaptable, and capable of protecting diverse populations against both existing and emerging infectious diseases.

